# Mucosal immunization with a delta-inulin adjuvanted recombinant spike vaccine elicits lung-resident immune memory and protects mice against SARS-CoV-2

**DOI:** 10.1038/s41385-022-00578-9

**Published:** 2022-11-21

**Authors:** Erica L. Stewart, Claudio Counoupas, Matt D. Johansen, Duc H. Nguyen, Stefan Miemczyk, Nicole G. Hansbro, Kia C. Ferrell, Anneliese Ashhurst, Sibel Alca, Caroline Ashley, Megan Steain, Warwick J. Britton, Philip M. Hansbro, Nikolai Petrovsky, James A. Triccas

**Affiliations:** 1 School of Medical Sciences, Faculty of Medicine and Health, The University of Sydney, Sydney, NSW Australia; 2 Tuberculosis Research Program at the Centenary Institute, The University of Sydney, Sydney, NSW Australia; 3 Sydney Institute for Infectious Diseases and the Charles Perkins Centre, The University of Sydney, Camperdown, NSW Australia; 4 Faculty of Science, School of Life Sciences, Centre for Inflammation, Centenary Institute and University of Technology Sydney, Sydney, NSW Australia; 5 Department of Clinical Immunology, Royal Prince Alfred Hospital, Sydney, NSW Australia; 6 Vaxine Pty Ltd., Bedford Park, Adelaide, 5042 SA Australia

## Abstract

Multiple SARS-CoV-2 vaccine candidates have been approved for use and have had a major impact on the COVID-19 pandemic. There remains, however, a significant need for vaccines that are safe, easily transportable and protective against infection, as well as disease. Mucosal vaccination is favored for its ability to induce immune memory at the site of infection, making it appealing for SARS-CoV-2 vaccine strategies. In this study we performed in-depth analysis of the immune responses in mice to a subunit recombinant spike protein vaccine formulated with the delta-inulin adjuvant Advax when administered intratracheally (IT), versus intramuscular delivery (IM). Both routes produced robust neutralizing antibody titers (NAb) and generated sterilizing immunity against SARS-CoV-2. IT delivery, however, produced significantly higher systemic and lung-local NAb that resisted waning up to six months post vaccination, and only IT delivery generated inducible bronchus-associated lymphoid tissue (iBALT), a site of lymphocyte antigen presentation and proliferation. This was coupled with robust and long-lasting lung tissue-resident memory CD4^+^ and CD8^+^ T cells that were not observed in IM-vaccinated mice. This study provides a detailed view of the lung-resident cellular response to IT vaccination against SARS-CoV-2 and demonstrates the importance of delivery site selection in the development of vaccine candidates.

## Introduction

In a display of collective scientific discovery and cooperation, numerous vaccine candidates against SARS-CoV-2 have been developed since the beginning of the COVID-19 pandemic. The unprecedented combined focus and injection of funds motivated by the pandemic led to multiple vaccines being approved by the World Health Organization (WHO) in record time.

mRNA vaccines, including candidates from Pfizer/BioNTech (USA/Germany) and Moderna (USA), were two of the first vaccines to be approved for emergency use by regulatory agencies after undergoing the full clinical trial schedule^1,2^. While the main advantage of this vaccine type is the ability for rapid manufacturing scale-up, the requirement for storage and transportation at ultralow temperatures is a barrier for access to rural or remote communities and increases distribution costs significantly^3^. Subunit vaccines are considered one of the safest vaccine formats, and are generally cheaper, easier to distribute, and cause less side-effects than their mRNA counterparts, but they require careful adjuvant selection lest the immune response be inappropriate or insufficient for a given pathogen^4^. The most advanced subunit vaccine candidates include Nuvaxovid/Covovax^TM^ and SpikoGen^TM^. The first is currently approved and being used in multiple countries including the UK and Australia, and the latter is approved and in use in Iran (NCT05005559).

Since the initiation of widespread vaccination schedules and the emergence of less virulent variants, there has been a reduction in the rate of COVID-19 related hospitalizations and severe COVID-19 cases^5^. However, vaccine-induced immunity has since been observed to wane by 6 months post immunization; this combined with the emergence of new variants has driven a decrease in vaccine effectiveness and a resurgence of COVID-19 transmission, although protection against severe disease has remained more robust^6,7^. This is especially the case for the recent Omicron variants, whereby neutralizing capacity of sera from vaccinated individuals is greatly reduced compared to against other variants^8^. Neutralizing antibodies (NAb) are currently the best correlate of protection against SARS-CoV-2 infection^9^. A limitation of NAb, however, is their reliance on their binding to tertiary structures, meaning they are more vulnerable to viral immune escape by mutations at the site of antibody binding^10^. In contrast, T cell recognition of SARS-CoV-2 remains robust against new variants^11^. Furthermore, as a protective factor against disease, cellular immune memory may be highly effective. Patients with agammaglobulinemia overcome COVID-19 infections without requiring ventilation, and early induction of T cell responses correlates with milder disease, suggesting a role for T cells in limiting the severity of SARS-CoV-2 infection^12,13^.

Mucosal vaccination is highly effective at generating local immune memory in the lungs, particularly resident memory T cells and IgA responses^14^. There are currently multiple intranasal SARS-CoV-2 vaccine candidates in Phase 3 clinical trials, including viral vector and protein subunit vaccines^15^. We have previously demonstrated that intrapulmonary vaccination with a subunit tuberculosis vaccine formulated with the particulate polysaccharide adjuvant Advax^TM^ generates robust resident memory T cells (TRMs) in the lungs along with systemic antibody responses^16^. The early pulmonary immune response to this vaccine is characterized by a broad chemotactic response that does not result in lasting inflammation^17^. While intranasal administration models are widely used, we selected the intrapulmonary route due to several reasons. Firstly, the mitigation of risk of damaging the orofacial nerve with vaccine induced inflammation as occurred after use of heat labile-toxin derived adjuvants^18^. Secondly, previous studies have shown that deposition of vaccine to the deep lungs can generate a stronger systemic immune response than intranasal immunization^19,20^. Murine intratracheal (IT) vaccines could translate readily into humans via aerosol delivery using a nebulizer, as was recently shown using the AdHu5Ag85A tuberculosis vaccine to induce TRMs in the lungs of healthy adults^21^. As interest in infectious respiratory diseases remains high, it is likely that there will be more similar studies performed in the future demonstrating the feasibility of this route.

The adjuvant in this vaccine, Advax, is a polysaccharide particulate adjuvant consisting of delta inulin particles of approximately 1–2 µm in diameter^22^. While the mechanism of action of Advax is still not fully understood, its major constituent, inulin, interacts with and activates the alternative complement pathway, and the immunogenicity of Advax is retained in inflammasome (NLRP3, Caspase-1 or IL-1r) knockout mice^23,24^. Advax has demonstrated a positive safety profile in both pre-clinical studies and clinical trials of multiple vaccines^25–27^. SARS-CoV-2 spike protein adjuvanted with Advax plus TLR-9 agonist CpG oligonucleotide prevented viral replication in the lungs and nasal cavity in a ferret and hamster infection models^28,29^. This vaccine, under the tradename SpikoGen^TM^, received emergency use authorization in Iran in early October 2021 based on positive clinical trial results^30,31^, thereby becoming the first recombinant spike protein vaccine to achieve this key milestone.

Here, we assessed the potential of IT delivery of recombinant spike protein formulated with Advax (Spike^Ax^) to generate durable local immunity in mice. In C57BL/6 mice, we demonstrated that IT Spike^Ax^ generates robust cellular memory in the lungs and bronchoalveolar lavage fluid (BALF), as well as sustained systemic NAb titers lasting up to six months post-vaccination. IM administered Spike^Ax^ also generated significant systemic NAb titers, but this was lower than that of IT immunization and was not associated with detectable lung memory T cell responses. In a K18-hACE2 mouse model, we demonstrated that both IT and IM delivery of Spike^Ax^ protected against clinical disease, providing sterilizing protection against homologous SARS-CoV-2 infection. Hence while vaccination with Spike^Ax^ via either delivery method provides robust protection against SARS-CoV-2, IT immunization generated lung-local immune memory as well as systemic antibody responses that were superior in magnitude and quality to that induced by parenteral administration.

## Results

### Intratracheal Spike^Ax^ immunization generates robust circulating cellular immune responses

**Fig. 1:**
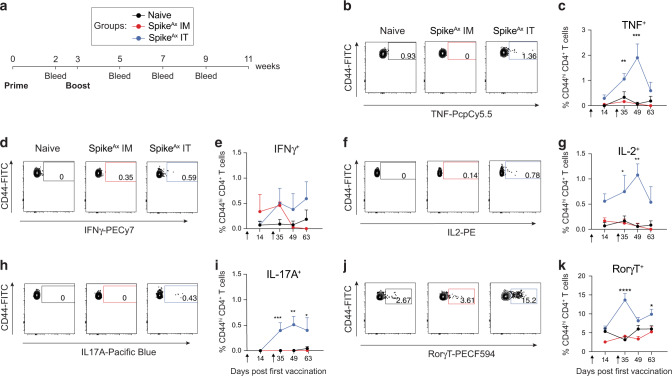
Spike^Ax^ IT vaccination promotes circulation of antigen-specific CD4^+^ T cells. C57BL/6 mice were vaccinated twice, three weeks apart, as per the schedule outlined in (**A**), or left naive, and bled at intervals after vaccination. Mice were vaccinated either IT or IM with 1 mg Advax/2 μg spike protein in PBS (Spike^Ax^) or with 2 μg spike protein in PBS (Supplementary Fig. 2). After each bleed, plasma was collected and PBMCs were isolated for flow cytometric analysis. PBMCs (**B**–**K**) were incubated in the presence of spike peptides for cytokine analysis. After restimulation PBMCs were stained for flow cytometry. Some PBMCs were stained without antigen recall for transcription factor analysis shown in (**J**) and (**K**). Graphs depict mean +/− SEM of four or five mice per group and are representative of more than two independent experiments, with arrows indicating time of vaccination. Statistical analysis of differences between Spike^Ax^ IT and naive mice were performed using a two-way ANOVA with Sidak’s multiple comparisons test, *p* < 0.05 (*), *p* < 0.005 (**), *p* < 0.0005 (***).

The administration route has a significant bearing on outcomes for many vaccine candidates^14^. Delivery to the peripheral lungs by IT (or intrapulmonary) administration, as opposed to the upper airways, enhances local and systemic antibody responses when compared to intranasal and parenteral vaccination^19,20^. We sought to compare the responses generated by Spike^Ax^ when administered by the IM versus IT routes. C57BL/6 mice were immunized twice either IM or IT with Spike^Ax^ or with spike protein alone, three weeks apart, then rested for eight weeks before analysis (Fig. 1A). Mice were bled at 2, 5, 7 and 9 weeks after the first immunization and peripheral blood mononuclear cells (PBMCs) were restimulated with immunogenic spike peptides for CD4^+^ and CD8^+^ T cells^32^, then stained intracellularly for cytokine expression. The gating strategy for identification of T cells and cytokine expression is depicted in Supplementary Fig. 1. Only Spike^Ax^ IT induced detectable CD4^+^ T cell responses in the blood, while CD8^+^ T cell cytokine responses were not detected in either vaccine group (data not shown). The observed Spike^Ax^ IT CD4^+^ T cell response was Th17-polarized, characterized by the expression of TNF, IL-2 and IL-17A up to six weeks after booster vaccination, with transient expression of RorγT (Fig. 1B–K). IL-10, GATA-3 and T-bet expression was not changed compared to naïve mice in any of the vaccinated groups (data not shown). Spike^Ax^ IM and spike protein alone control groups (Supplementary Fig. 2) did not generate detectable circulating anti-spike CD4^+^ T cell responses.

### Spike^Ax^ promotes long lasting resident T cells in the lungs and airways after IT vaccination

**Fig. 2:**
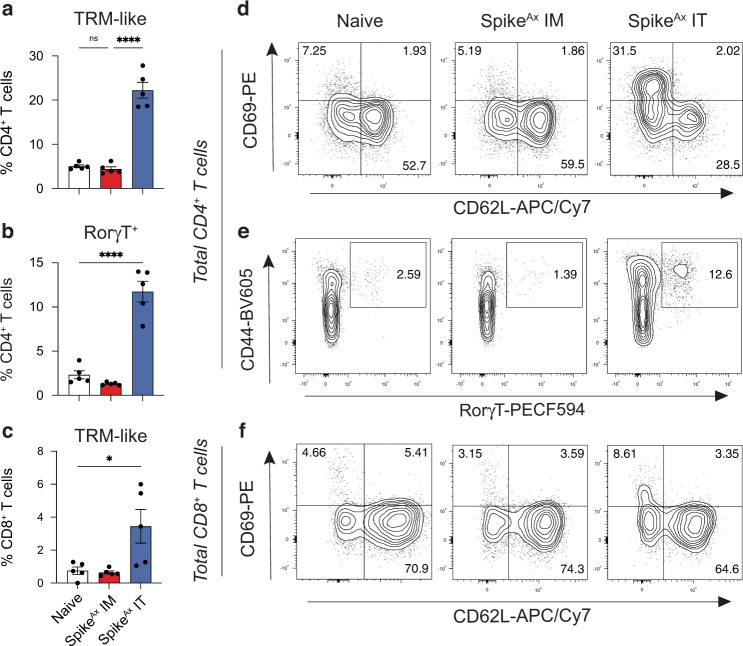
Spike^Ax^ IT vaccinated mice retain lung-resident memory T cells up to eight weeks post-vaccination. C57BL/6 mice were vaccinated as in Fig. 1 and at eight weeks post-boost mice lungs were collected for flow cytometric analysis. **A** Percentage of total CD4^+^ T cells in the lungs expressing TRM-like phenotype (CD44^+^CD69^+^CD62L^−^). **B** Percentage of total CD4^+^ T cells in the lungs expressing RorγT. **C** Percentage of total CD8^+^ T cells in the lungs expressing TRM-like markers (CD44^+^CD69^+^CD103^+^CD62L^−^). **D**, **E** Representative FACS plots of lung CD4^+^ T cells. **F** Representative FACS plots of lung CD8^+^ T cells. Bar graphs (**A**–**C**) depict individual and mean values +/− SEM of five mice per group and are representative of more than two independent experiments. Statistical analysis of differences between groups was performed using a one-way ANOVA with Tukey’s multiple comparisons test, *p* < 0.05 (*), *p* < 0.005 (**), *p* < 0.0005 (***). FACS plots (**D**–**F**) are representative of the mice shown in graphs (**A**–**C**).

**Fig. 3:**
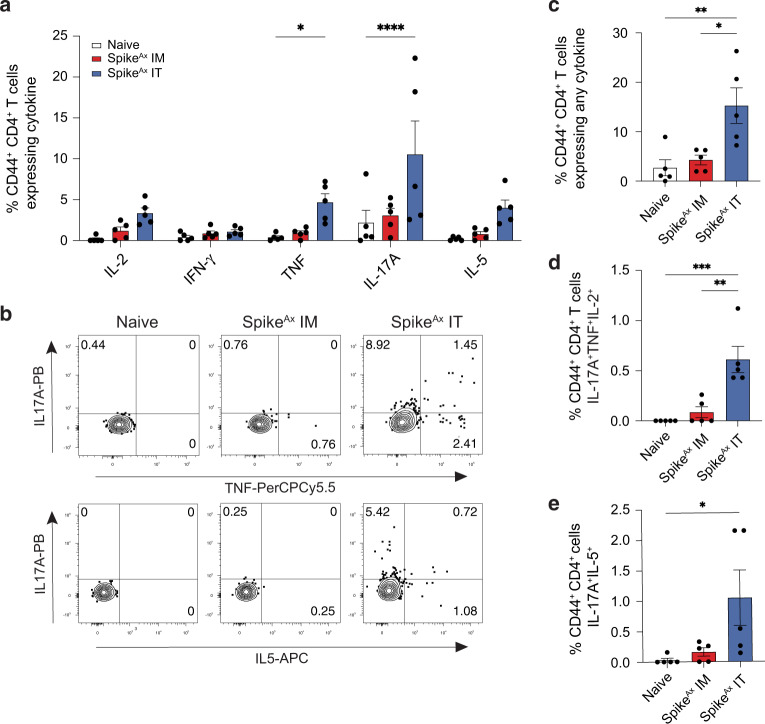
Spike^Ax^ IT vaccinated mice generate lasting antigen-specific Th17 and Th2 cells in the lungs. C57BL/6 mice were vaccinated as in Fig. 1 and at eight weeks post boost lungs were collected for flow cytometric analysis. Single-cell suspensions of lungs were restimulated with spike peptides and eBioscience protein transport inhibitor cocktail. Following restimulation, the production of cytokines (IL-2, IFN-γ, TNF, IL-17A, IL-5, and IL-10 (not shown)) by CD4^+^ T cells was determined by flow cytometry (**A**, **B**) as per the gating strategy in Supplementary Fig. 1. Co-expression of cytokines was measured by Boolean gating in FlowJo software (**C**–**E**). Bar graphs (**A**, **C**–**E**) depict individual and mean values +/− SEM of five mice per group and are representative of more than two independent experiments. Statistical analysis on difference between groups was performed using a two-way ANOVA (**A**) or one-way (**C**–**E**) ANOVA with Tukey’s multiple comparisons test, *p* < 0.05 (*), *p* < 0.005 (**), *p* < 0.0005 (***). FACS plots (**B**) are representative of the mice shown in graphs.

One of the primary advantages of mucosal vaccination is the induction of local immune memory that can rapidly respond to microbial challenge^33^. In accordance with the responses in the blood, Spike^Ax^ IT generated a strong and lasting memory T cell response in the lungs. Conversely, Spike^Ax^ IM and spike alone controls did not produce a tissue-resident memory (TRM)-like cellular response (defined by CD44^+^CD69^+^CD62L^−^CD4^+^ T cells or CD103^+^CD44^+^CD69^+^CD62L^−^ CD8^+^ T cells in the lungs^33^) compared to naïve animals (Fig. 2, Supplementary Fig. 3). CD4^+^ (Fig. 2A, D) and CD8^+^ (Fig. 2C, F) TRM-like cells were significantly increased in the lungs of Spike^Ax^ IT animals but not Spike^Ax^ IM mice. In IT-vaccinated mice, CD4^+^ TRM-like cells displayed a Th17 phenotype identified by higher expression of RorγT (Fig. 2B, E), with no differences in the expression of the Th1 transcription factor, T-bet (data not shown). CD8^+^ TRM-like cells displayed no discernable polarization and did not express higher levels of RorγT or T-bet (data not shown). The percentage of antigen-specific, cytokine-producing CD4^+^ T cells in the lungs of the Spike^Ax^ IT group was significantly higher than that of both naïve and Spike^Ax^ IM mice, and aligned with systemic PBMC data (Fig. 3A, C). Boolean gating to identify multifunctional cytokine-expressing cells revealed antigen-specific CD4^+^ T lymphocytes from the Spike^Ax^ IT group expressed significantly higher levels of IL-17A and TNF in the lungs, with not significant levels of IL-5 and IL-2 (Fig. 3A). The presence of both IL-5- and IL-17A-producing T cells suggested the possible presence of Th2/Th17 cells, but while there was some co-expression of IL-17A and IL-5 in Boolean gating (Fig. 3B, E), most CD4^+^ T cells expressed a single cytokine with few multifunctional CD4^+^ T cells observed (data not shown). More consistent was the presence of Th17 cells characterized by concurrent IL-17A, TNF, and IL-2 expression (Fig. 3B, D). Despite restimulating lung cells with peptides considered immunogenic for both CD4^+^ and CD8^+^ T cells, there was no detectable CD8^+^ T cell cytokine response observed (data not shown).

**Fig. 4:**
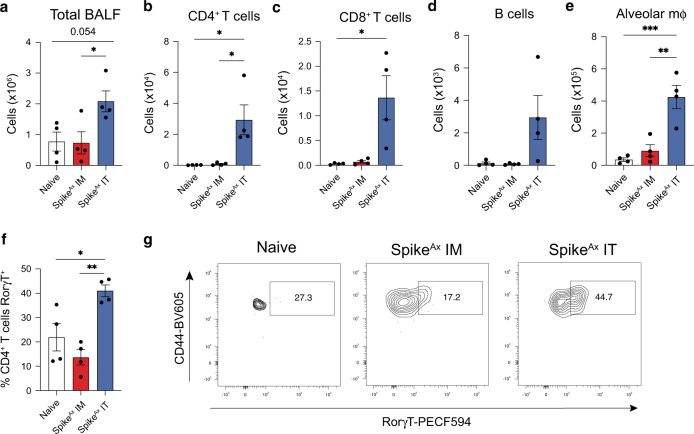
Spike^Ax^ IT induces lasting lymphocytic recruitment to the airways. C57BL/6 mice were vaccinated as in Fig. 1 and at eight weeks after the final vaccination the bronchoalveolar lavage fluid (BALF) was collected for antibody and flow cytometric analysis; intravascular staining was performed to exclude circulating cells. **A** Total BALF cell number. **B**–**E** Total number of CD4^+^ T cells, CD8^+^ T cells, B cells, and alveolar macrophages in the BALF. **F**–**G** Percentage of CD4^+^ T cells in the BALF expressing RorγT. Bar graphs show individual values of four mice per group +/− SEM and are representative of more than two independent experiments. FACS plots are representative of more than two independent experiments. The significance between groups was determined via a one-way ANOVA with Tukey’s multiple comparisons test, *p* < 0.05 (*), *p* < 0.005 (**), *p* < 0.0005 (***).

TRMs in the lungs are associated with more rapid immune responses and better protection against pulmonary pathogens, as is the presence of lymphocytes in the alveolar compartment^34,35^. To determine if IT immunization with Spike^Ax^ generated airway lymphocytes and antibodies, we collected the bronchoalveolar lavage fluid (BALF) at the time of sacrifice eight weeks post-booster vaccination. Cells were gated as intravascular negative prior to analysis to ensure there was no contamination of blood lymphocytes in the BALF (Supplementary Fig. 1E). The BALF of IT-vaccinated animals contained higher cell numbers compared to both naïve and Spike^Ax^ IM vaccinated mice, while spike protein alone generated no significant changes in cell number (Fig. 4A, Supplementary Fig. 4). The enriched cellular composition was made up of various leukocytes, including CD4^+^ and CD8^+^ T cells, B cells and alveolar macrophages, all of which were observed in the BALF of IT immunized mice at significantly higher numbers than in other groups (Fig. 4B–E). Phenotypically, CD4^+^ T cells in the BALF displayed a Th17 TRM phenotype, defined by the expression of the Th17 master regulator RorγT (Fig. 4F, G), the tissue residency marker CD69 and lack of CD62L (data not shown)^16^. Alveolar macrophages in the Spike^Ax^ IT vaccinated animals displayed reduced Siglec-F expression (Supplementary Fig. 4H), indicative of the possible induction of monocyte-derived alveolar macrophages, which are capable of enhanced microbial killing^36^. T cell phenotypes in the draining lymph nodes were similar to those observed in the blood and the lungs (Supplementary Fig. 5). Thus, despite administering the same vaccine IT and IM, only Spike^Ax^ IT immunized animals generated a robust and lasting T cell response both systemically and locally in the pulmonary tissue. This T cell response was characterized by primarily a Th17 phenotype, with a moderate Th2 response also observed.

### Spike^Ax^ IT immunization generates iBALT and higher antibody titers in the blood and BALF

**Fig. 5:**
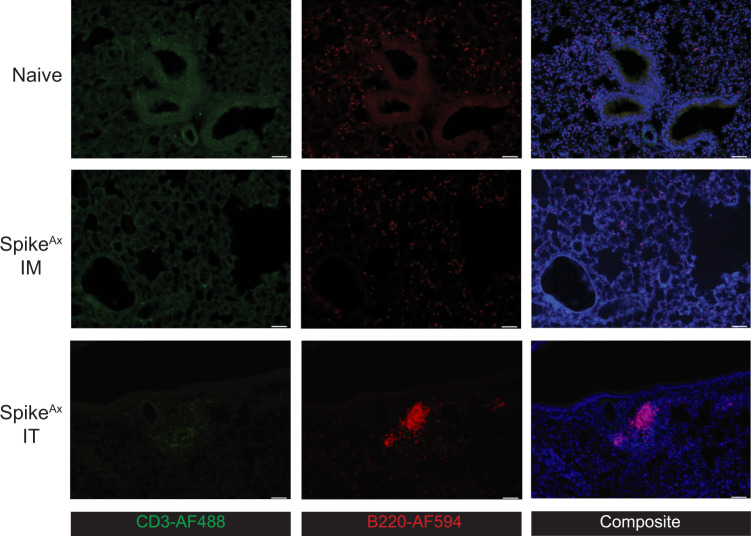
IT vaccination with Spike^Ax^ generates lasting pulmonary iBALT structures. C57BL/6 mice were vaccinated as in Fig. 1 and at eight weeks post boost lung lobes were collected for imaging and stained with anti CD3-AF488 (green), anti B220-AF594 (red) and DAPI (blue). Scale bars depict 50 μm in length. Images are representative of four mice from a single experiment.

Mucosal vaccination to the lungs as well as infection with pulmonary pathogens has been observed to generate lymphocytic aggregations near the airways known as inducible bronchus-associated lymphoid tissue (iBALT), which can act as a site of antigen presentation and lymphocyte proliferation^37^. Immunofluorescence staining for CD3, B220, and DAPI revealed distinct aggregates of B and T lymphocytes near major airways in the Spike^Ax^ IT group eight weeks after the final immunization that were absent in naïve, spike alone and Spike^Ax^ IM mice (Fig. 5; Supplementary Fig. 6). The presence of such iBALT structures is associated with faster lung-local antibody production at the time of microbial challenge^37^.

**Fig. 6:**
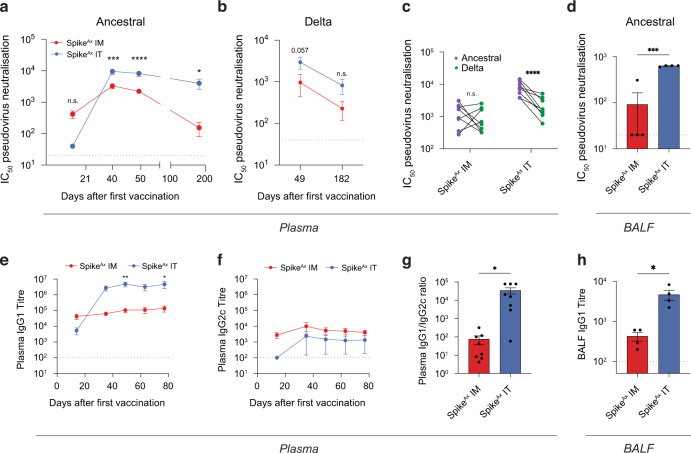
Vaccination with Spike^Ax^ IT produces lasting circulating and lung-local neutralising antibodies and IgG1. C57BL/6 mice were vaccinated as in Fig. 1 and at time points after each vaccination, plasma was collected and assessed for neutralizing antibody titers (NAb) against pseudovirus expressing the ancestral (**A**) or delta spike proteins (**B**). **C** Individual animal comparisons of NAb at four weeks post booster (day 49). **D** NAb titer in bronchoalveolar lavage fluid (BALF) taken eight weeks after booster vaccination (day 77 after first immunization). Plasma was also tested for the presence of spike-specific IgG1, IgG2c via ELISA (**E**, **F**). **G** Plasma IgG1/IgG2c ratios were calculated for day 49 samples. **H** BALF was tested for spike-specific IgG1. Dotted lines depict the limit of detection of assays. Graphs depict pooled or representative mean values +/− SEM of two experiments with four or five mice per group, except for (**D**) and (**H**) which are representative of a single experiment. For line graphs, statistical analysis on differences between Spike^Ax^ IT and Spike^Ax^ IM were performed using a two-way ANOVA with Holm-Sidak multiple comparisons test (**A**–**C**, **E**–**F**), *p* < 0.05 (*), *p* < 0.005 (**), *p* < 0.0005 (***). For bar graphs, an unpaired two-tailed T-test was performed, *p* < 0.05 (*), *p* < 0.005 (**), *p* < 0.0005 (***).

NAb are the most important correlate of the potential effectiveness of SARS-CoV-2 vaccine candidates^9,38^. Two weeks after the initial prime vaccination, Spike^Ax^ IM generated significant NAb titers against pseudovirus expressing ancestral spike, and these were approximately doubled after booster vaccination (Fig. 6A). IT administration generated lower, almost undetectable titers compared to IM after the prime vaccination. After boosting however, the NAb titers of IT-vaccinated animals increased up to 100-fold and were significantly higher than those in Spike^Ax^ IM mice, and these titers were maintained for up to six months after vaccination (Fig. 6A). IT administration also generated NAbs in the airways that were not observable after IM immunization (Fig. 6D). Both modes of administration were effective at generating antibodies capable of neutralizing pseudovirus expressing the delta variant spike protein (Fig. 6B, C). Thus, while both administration routes induced significant NAbs, IT vaccination generated increased levels of circulating and airway NAbs, that lasted up to six months post-booster vaccination.

Spike^Ax^ IT also generated a greater spike-specific antibody response than Spike^Ax^ IM. This antibody response was primarily composed of IgG1 antibodies, with little to no IgG2c, and moderate spike-specific IgA that was not observed in IM immunized mice (Fig. 6E–G, Supplementary Fig. 7A). IgG1 titers were also detected in the BALF of Spike^Ax^ IT animals at significantly higher levels than Spike^Ax^ IM animals, and while not significant, there was also a trending increase in spike-specific IgA in the BALF of Spike^Ax^ IT animals (Fig. 6H, Supplementary Fig. 7B). While Spike^Ax^ IM animals had reduced IgG1 responses, they did produce greater IgG2c than Spike^Ax^ IT (Fig. 6F, G). Spike-alone controls generated little to no detectable spike-specific antibodies after IT administration and highly variable binding antibodies and NAb after IM delivery that were completely abrogated by six months (Supplementary Fig. 2). Overall, immunization with Spike^Ax^ was effective at producing robust systemic IgG responses irrespective of immunization route, however IT vaccination produced greater and longer lasting systemic and lung-localized NAb and IgG responses and pulmonary T cell responses.

### Spike^Ax^ vaccine is protective against SARS-CoV-2 infection in a K18-hACE2 mouse model when administered IT or IM

**Fig. 7:**
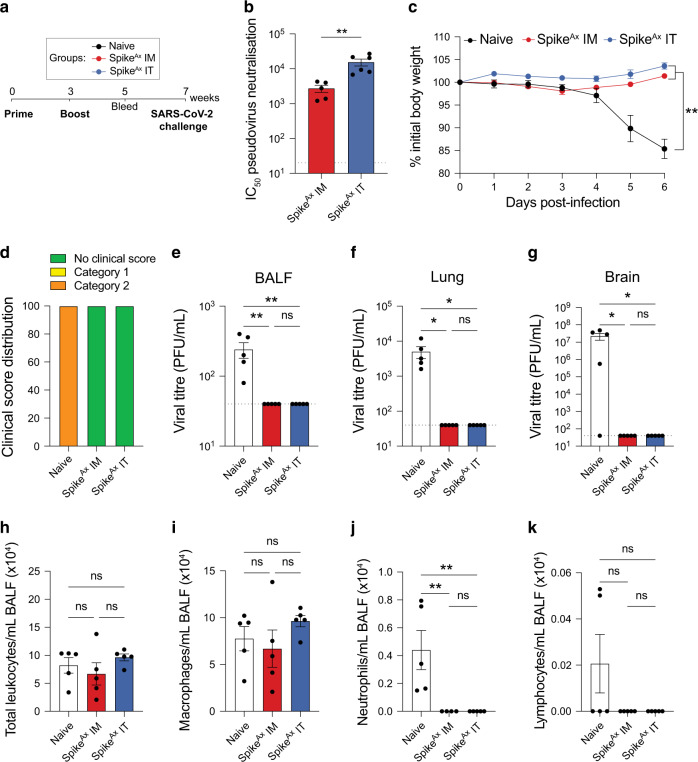
Vaccination with Spike^Ax^ either IT or IM provides sterilizing immunity against SARS-CoV-2 infection. K18-hACE2 mice were vaccinated as described in Fig. 1. Blood samples were collected two weeks after the final immunization, and mice were inoculated intranasally with 1000 PFU SARS-CoV-2 (VIC01/2020) at four weeks (**A**). Neutralizing antibody titres (NAb) against ancestral SARS-CoV-2 were determined from plasma taken two weeks after the final immunization (**B**). Mice were monitored and weighed daily after SARS-CoV-2 infection, until reaching experimental endpoint at day 6 (**C**). Clinical scores of mice at day 6 post infection (**D**). Viral titres in BALF (**E**), lungs (**F**), and brain (**G**) were determined using plaque assays; dotted lines indicate the limit of detection. Numbers of total leukocytes (**H**), macrophages (**I**), neutrophils (**J**), and lymphocytes (**K**) in the BALF were determined using a haemocytometer and cytospin. Graphs depict a single experiment with 5 mice per group. For (**B**), statistical analysis was performed using an unpaired two-tailed T-test, *p* < 0.05 (*), *p* < 0.005 (**). For (**C**) a two-way ANOVA was performed with post-hoc Tukey test to correct for multiple comparisons, *p* < 0.05 (*), *p* < 0.005 (**). For (**E**–**K**) a one-way ANOVA with post-hoc Tukey test was performed, *p* < 0.05 (*), *p* < 0.005 (**).

To determine if the presence of NAb and spike-specific T cells induced by Spike^Ax^ correlated with protection against SARS-CoV-2 infection, K18-hACE2 mice were immunized with Spike^Ax^ via the IT or IM route, or left naïve as controls (Fig. 7A). As was observed in C57BL/6 mice, IT administration led to greater plasma NAb than IM delivery, although both delivery methods led to significant circulating NAb (Fig. 7B). Four weeks after the last vaccination, mice were infected intranasally with 1000 PFU ancestral variant SARS-CoV-2 (VIC01/2020) and assessed and monitored for six days before determination of viral load, as previously described^39,40^. Spike^Ax^ vaccinated mice, both via IM or IT route, were completely protected from the significant weight loss (versus approximately 15% weight loss seen in control animals) and the clinical features observed in challenged unvaccinated animals (Fig. 7C, D). Furthermore, while unvaccinated mice displayed significant viral titers in the BALF, lung and brain, there was no detectable viral load in any of these sites in vaccinated animals (Fig. 7E–G). No concerns were raised regarding the possibility of immunopathology due to pulmonary vaccine delivery, with no difference in the number of leukocytes, macrophages, neutrophils or lymphocytes in the BALF of mice immunized via the IT or IM route (Fig. 7H–K). This contrasted with an increase in neutrophils and lymphocytes observed in the BALF of unvaccinated mice (Fig. 7J, K). Increased eosinophils were not detected in the BALF of any of the infected animals (data not shown). Thus, irrespective of the delivery approach used, Spike^Ax^ vaccine provided robust protection against SARS-CoV-2 infection in a mouse challenge model without any associated immunopathology.

## Discussion

The SARS-CoV-2 pandemic has taken millions of lives and significantly altered countless others. Antiviral treatments against the infection remain limited, so there is an urgent requirement for more effective vaccines. While some vaccines are currently in use around the world, there is a need for a variety of vaccine options, especially for booster immunization^3^. There is also a great need for affordable vaccines that are amenable to distribution to rural and remote areas^3^. Here, we investigated the impact of delivery route on the immunogenicity and protective efficacy of recombinant spike antigen formulated with Advax adjuvant (Spike^Ax^).

A primary differentiating outcome of IT vaccination, when compared to parenteral vaccination, is the generation of a lung-localized memory immune response, including resident memory T cells and iBALT^16,37,41^. We observed both immune features with Spike^Ax^ and, furthermore, identified that IT administered Spike^Ax^ generated a robust circulating CD4^+^ T cell response with a Th17 phenotype characterized by the expression of RorγT and secretion of IL-17A (Fig. 1), that was not present in the Spike^Ax^ IM group. Advax is unique as an adjuvant in that it is not highly polarizing when administered alone^42^. T cell polarization can be influenced by the choice of antigen or by the addition of polarizing agents, such as CpG that is strongly Th1 polarizing and used as Advax-CpG adjuvant in both the pre-clinical trials of Covax-19 vaccine^28^, and the SpikoGen^TM^ vaccine approved for emergency use in Iran (NCT05005559)^30^. We observed moderate, but not significant, IFN-γ expression by circulating vaccine-specific CD4^+^ T cells but little to no IFN-γ expression in the lungs (Figs. 1, 3). A previous study by our group demonstrated that Advax-CpG formulated with a tuberculosis vaccine enhanced CD4^+^ IFN-γ and IL-17A responses after challenge, but the CpG component was dispensable for protective efficacy^16^. CpG has also been used as a Th1-polarizing TLR agonist in several mucosal SARS-CoV-2 vaccine candidates, thus, future investigation of Advax-CpG in a pulmonary SARS-CoV-2 vaccine may be of interest^43,44^.

Early induction of SARS-CoV-2-specific CD4^+^ and CD8^+^ T cells is associated with more rapid viral clearance and milder disease, and T cells alone can partially protect SARS-CoV-2-infected mice from severe disease^13,32^. IT Spike^Ax^ vaccination generated increased proportions of CD8^+^ T cells expressing resident memory markers (CD44, CD103, and CD69 in the absence of CD62L) in the lungs (Fig. 2). Greater numbers of airway CD8^+^ T cells were also observed in the BALF up to eight weeks post-vaccination (Fig. 4). Depletion of CD8^+^ T cells in convalescent rhesus macaques partially reduced protection against SARS-CoV-2 provided by prior infection, and vaccine-induced CD8^+^ T cells were protective in the absence of antibodies in two independent studies^45–47^. While we observed greater numbers of TRM-like CD8^+^ T cells in the airways and lungs of IT-immunized animals, we did not observe significant cytokine expression after restimulation of lung cells with MHC-I restricted peptides (data not shown), but we did not test for evidence of antigen-specific cytotoxicity such as staining for granzymes and perforins. It is hypothesized that Advax may promote the recruitment of APCs capable of cross-presentation, although the exact mechanism for its enhancement of CD8^+^ T cell responses requires further investigation^24^. While the functionality of the lung-resident CD8^+^ T cells induced by IT Spike^Ax^ is not defined by this study, it seems likely that given their specific induction in vaccinated groups and their anatomical location that they may contribute to the observed protection against SARS-CoV-2 infection.

IT Spike^Ax^ vaccination generated increased CD4^+^ T cells with a Th17 TRM-like phenotype in the lungs eight weeks after vaccination (Figs. 2, 3). In infected individuals, pathologic Th1 and Th17 cells are implicated as contributing to SARS-CoV-2-induced hyperinflammation and acute respiratory distress syndrome, while excess Th2 cells can be associated with pulmonary allergic inflammation and vaccine-associated enhanced disease^48–50^. Multiple SARS-CoV-2 vaccine candidates have been tested intranasally and found to induce sterilizing immunity^51–53^. A recent study by Kingstad-Bakke et al. described robust Th1/Th17 TRM generation after intranasal administration of two adjuvanted vaccines and demonstrated a significant role for vaccine-induced CD4^+^ T cells in protection against homologous infection^43^. Despite the many studies of mucosal SARS-CoV-2 vaccines, however, few have measured IL-17A so the contribution of memory Th17 cells to protection against SARS-CoV-2 is unclear. In mucosal vaccination studies against other pathogens, lung resident memory Th17 cells are capable of enhancing early IFN-γ expression as well as displaying plasticity in their cytokine expression in response to infection^54,55^. While increased antigen-specific Th17 cells in the blood and lungs have been correlated with severe COVID-19 disease, we did not observe neutrophilia or eosinophilia in IT-immunized animals despite the induction of Th2 and Th17 polarized TRMs^49,56^. In fact, IT immunization with Spike^Ax^ was highly protective against SARS-CoV-2 infection, with no discernable immunopathology observed (Fig. 7).

We also observed low but significant induction of Th2-polarized CD4^+^ T cells (Fig. 3). Th2 cells in the lungs are traditionally associated with asthma and allergy and have been associated with vaccine-enhanced disease in influenza and RSV, although their role in SARS-CoV-2 infection remains unclear. Despite the potential pathological roles of Th2 and Th17 cells, after challenge, Spike^Ax^ IT immunized mice were well protected against SARS-CoV-2 infection and did not display any clinical signs of disease or eosinophilia (Fig. 7). It is possible that the role of CD4^+^ TRMs generated by Spike^Ax^ IT is to assist in the production of local protective immune responses. It has been observed that the magnitude of anti-SARS-CoV-2 IgG and IgA is correlated with virus-specific CD4^+^ T cell responses^38,57^. A recent study by Afkhami et al. also illustrated a role for T cells in inducing protective, trained alveolar macrophages after intranasal administration of a trivalent adenoviral-vectored vaccine^58^. However, given the pathogenic role of Th2 and Th17 cells in some contexts, future studies of Spike^Ax^ IT may involve depletion of vaccine-induced CD4^+^ T cells, or their associated cytokines, during challenge to determine the mechanistic role of these cells in the protective efficacy provided by Spike^Ax^ IT.

Antibodies, particularly NAbs, are an essential component of protective immune responses against SARS-CoV-2^9^. Both immunization strategies generated significant NAb titers in the plasma against pseudoviruses expressing either ancestral or delta variant spike protein (Fig. 6). However, IT administration produced a more robust humoral response that included higher sera NAb than IM administration, as well as the presence of NAb in the airways and a spike-specific IgA response that was not present in IM immunized animals (Fig. 6, Supplementary Fig. 7). Confocal imaging of the lung tissue revealed lymphoid aggregates, known as iBALT, adjacent to major airways in IT vaccinated animals (Fig. 5). iBALT is a lung-local site for the induction of adaptive immune memory, whereby antigen presentation and lymphoid proliferation is initiated more rapidly and without the need for trafficking to the local lymph node^37^. These lung-resident sites of lymphoid proliferation may be responsible for the lung-localised NAb we observed in IT-immunized animals. A significant concern regarding currently used SARS-CoV-2 vaccines is the waning of NAb from five months after immunization, corresponding with reduced effectiveness^6,7^. IT vaccination generated longer-lasting NAb than parenteral immunization; at six months post-booster, neutralizing activity against ancestral and delta pseudoviruses in the plasma of IT-vaccinated animals remained robust, while the NAb of IM-immunized animals began to wane (Fig. 6). It is unclear why there was the observed difference in the maintenance of circulating NAb between administration routes. It is possible that lung-resident iBALT structures continue to produce NAb systemically, or that the inflammatory nature effect of pulmonary administration itself leads to a more pronounced T-helper cell phenotype that enhances humoral responses^43,59,60^. The mechanism responsible requires further examination. The presence of high NAb titers strongly correlates with protection from disease in hamsters and ferrets^9^, and in line with this we observed both routes of vaccine delivery generated sterilizing immunity against SARS-CoV-2 challenge (Fig. 7). IT administration, however, provided the additional protection of NAb in the airways, the site of SARS-CoV-2 infection, and also produced longer-lasting NAb than parenteral administration.

This study demonstrates that IT administration of Spike^Ax^ vaccine induced prominent lung-resident cellular immune responses defined by the presence of pulmonary CD4^+^ and Th17 TRMs, B cells and BALF antibodies, and robust humoral immune responses. We have established that Advax, administered via either route, generates robust NAb responses and sterilizing immunity against SARS-CoV-2 infection, and is a suitable adjuvant candidate for both pulmonary and parenteral vaccines. Further investigations into the contribution of cellular immunity, however, are required for a full understanding of the mechanisms of vaccine-induced protective immunity against SARS-CoV-2.

## Materials and methods

### Mice and vaccinations

Male C57BL/6 mice 6–8 weeks of age were sourced from Animal Resources Centre (Perth, Western Australia), and male hemizygous K18-hACE2 mice on a C57BL/6 background were bred and maintained in specific-pathogen-free conditions at the Centenary Institute (Sydney, New South Wales). Mice were chosen at random for experimental groups of four to five mice per group. All mouse experiments were approved by the Sydney Local Health District Animal Ethics and Welfare Committee (protocol 2020-009 and 2020-019). SARS-CoV-2 spike protein was produced in a recombinant baculovirus system as described previously^28^. The Advax adjuvant (delta-inulin, 50 mg/mL) was supplied by Vaxine Pty Ltd. Mice were vaccinated IM in the hind leg following sedation with isoflurane and injection of 2 µg spike protein and 1 mg Advax or 2 µg spike protein alone in a total volume of 100 µL of endotoxin-free PBS (Sigma-Aldrich, NSW). For IT vaccinations, mice were anaesthetized by intraperitoneal injection with ketamine (80 mg/kg) and xylazine (10 mg/kg) in PBS followed by intratracheal installation with 2 µg spike protein and 1 mg Advax or 2 µg spike protein alone in a total volume of 50 µL in endotoxin-free PBS (Sigma-Aldrich, NSW) using the PennCentury Microsprayer Aerosoliser (PennCentury, PA, USA).

### Blood sample collection and analysis

Blood samples were collected into tubes containing 10 µL heparin (50 U, Sigma-Aldrich, NSW), centrifuged to separate plasma and PBMCs isolated using Histopaque 1083 (Sigma-Aldrich, NSW). For antigen recall, PBMCs were restimulated with spike peptides identified to be immunogenic for CD4^+^ and CD8^+^ T cells respectively in C57BL/6 mice by Zhuang et al.^32^, spike peptide 62–76 at 6 µM concentration and spike peptide 538–546 at 5 µM concentration. Cells were then incubated at 37 °C for five hours in the presence of Protein Transport Inhibitor Cocktail (Life Technologies, Thermo Fisher Scientific, NSW). PBMCs were stained for intracellular cytokine production (as described below). Alternatively, unstimulated PBMCs were stained intracellularly immediately after collection for transcription factor expression (described below).

### Preparation of samples for flow cytometry

Lungs and lymph nodes were processed as described previously^17^. Intravascular staining was performed by the intravascular injection of 15 µg/mL CD45-biotin (clone 30-F11, BioLegend, CA, USA) three minutes before euthanasia. BALF was collected by tracheal intubation followed by flushing of the lungs with 1 mL cold PBS. Cells were centrifuged out of the BALF for flow cytometric analysis and BALF kept for antibody analysis. For flow cytometric analysis, single cell suspensions were resuspended in 2% FCS, 5 mM EDTA in PBS and pre-incubated with Purified Rat Anti-Mouse CD16/CD32 clone 2.4G2 (Becton Dickinson, NSW), Fixable Blue Dead Cell stain (Life Technologies, Thermo Fisher Scientific, NSW) and streptavidin conjugated to PE/Cy7 (Becton Dickinson, NSW). Single-cell suspensions were then incubated for 30 min with fluorochrome labelled monoclonal antibodies outlined in Supplementary Table 1. For intracellular cytokine staining, cells were then permeabilized and fixed using the BD Cytofix/Cytoperm Fixation/Permeabilization Kit (Becton Dickinson, NSW) followed by incubation with fluorescently conjugated monoclonal antibodies. For intracellular transcription factor staining, cells were permeabilized and fixed using the eBioscience Foxp3/Transcription Factor Staining Buffer Set (Thermo Fisher Scientific, NSW) followed by incubation with monoclonal antibodies. Samples were analyzed on a BD LSR-II cytometer (Becton Dickinson, NSW) at the Sydney Cytometry Facility (Charles Perkins Centre, NSW). Flow cytometric data was analyzed using the gating strategies outlined in Supplementary Fig. 1.

### Immunofluorescence staining

Lung lobes were prepared for immunofluorescence staining as described previously^16^. Briefly, lungs were perfused and the left lobe was removed and inflated with 4% paraformaldehyde (Thermo Fisher Scientific, NSW). After fixation, lobes were incubated in 15% sucrose solution, followed by 30% sucrose solution. Once saturated, lungs were snap frozen at −80 °C in Optimal Cutting Temperature Compound (VWR Chemicals, Singapore). Lobes were sectioned using a Cryotome E (Thermo Fisher Scientific, NSW). For staining, slides were washed in PBS and blocked using 3% Normal Goat Serum (Sigma-Aldrich, NSW) and 0.1% Triton-X 100 (Sigma-Aldrich, NSW) in PBS. Slides were stained with AF488-conjugated anti-mouse CD3 (Clone 17A2, Biolegend, CA, USA), AF594-conjugated anti-mouse B220 (clone RA3-6B2, Biolegend, CA, USA) and NucBlue Fixed Cell ReadyProbes Reagent (Life Technologies, Thermo Fisher Scientific, NSW). Samples were then mounted using Prolong Diamond Antifade Mountant (Thermo Fisher Scientific, NSW) and imaged using a BX51 microscope (Olympus, Japan).

### Antibody ELISAs

To analyze plasma and BALF samples for the presence of antibodies against spike protein, Corning 96 Well Clear PVC Assay Microplates (Sigma-Aldrich, NSW) were coated with spike protein (1 µg/mL) overnight at room temperature (RT). Plates were then blocked for two hours with 3% Bovine Serum Albumin (BSA) (Moregate, QLD) in PBS at RT, followed by washing with 0.1% BSA, 0.01% Tween in PBS. Plasma or BALF samples were serially diluted in 1% BSA in PBS then added to plates and incubated at for 45 min at 37 °C. After washing, detection antibodies were added at their required concentrations (Goat Anti-Mouse IgG1 heavy chain (Biotin) (Abcam, Vic), 1:50,000; Goat Anti-Mouse IgG2c heavy chain (Biotin) (Abcam, Vic), 1:10,000; Goat Anti-Mouse IgA alpha chain (Biotin) (Abcam, Vic), 1:50,000) and incubated for one hour at RT. Plates were again washed followed by the addition of streptavidin-HRP (Streptavidin (HRP) (Abcam, Vic), 1:30,000) and incubated for 30 min at RT. After a final wash, antibody binding was visualized by the addition of substrate (0.1 mg/mL 3,3′,5,5′-Tetramethylbenzidine (Sigma-Aldrich, NSW)) and hydrogen peroxide in 0.5 M phosphate citrate buffer (Sigma-Aldrich, NSW). Absorbances were read at 450 nm using the M1000 pro plate reader (Tecan, Mannedorf, Switzerland). Titers were determined using GraphPad Prism 9 software (GraphPad, California, USA) to fit a sigmoidal curve and calculate the intersection with three standard deviations above the mean negative control value (average absorbances of unvaccinated mouse plasma or BALF).

### Pseudovirus entry neutralization assay

Pseudovirus particles expressing spike proteins were produced as described previously with plasmids expressing either ancestral or delta spike proteins^61,62^. To measure neutralizing capacity of plasma and BALF, HEK-293 cells transduced to express ACE-2 were seeded onto CellCarrier-384 Ultra Microplates, PDL coated (PerkinElmer, Vic), and incubated overnight. The next day, plasma or BALF was diluted in DMEM media (Life Technologies, Thermo Fisher Scientific, NSW) and incubated at 37 °C for 1 h in the presence of pseudovirus. After incubation, cells were spinoculated (1 h, 35 °C, 800 × *g*) with virus and antibody then left for 72 h at 37 °C. Cells were then fixed in freshly prepared 4% paraformaldehyde (Life Technologies, Thermo Fisher Scientific, NSW) followed by nuclear staining with DAPI (Life Technologies, Thermo Fisher Scientific, NSW). Cells were analyzed for GFP fluorescence using an Opera Phenix High-Content Screening System (PerkinElmer, Vic) and Harmony® high-content analysis software (Perkin Elmer, Vic) provided by the Sydney Cytometry Facility (Charles Perkins Centre, NSW).

### SARS-CoV-2 challenge

Hemizygous male K18-hACE2 mice were immunized IT or IM with recombinant SARS-CoV-2 spike protein mixed with Advax adjuvant as described above or remained unimmunized. Mice were immunized twice, three weeks apart, and then rested for four weeks before being transferred to the PC3 facility in the Centenary Institute for SARS-CoV-2 infection. Mice were anaesthetized using isoflurane prior to intranasal challenge with SARS-CoV-2 (1,000 PFU, ancestral variant VIC01/2020) in 30 µL total volume, as we have described previously^39,40^. Mice were weighed and monitored for clinical symptoms daily, with increased monitoring after the observation of symptomatic disease. Clinical scores were determined based on a combination of weight loss, hunching, ruffling, isolation in the cage, lethargy, and labored breathing. Mice were euthanized at day 6 post-infection and viral load was determined in the BALF, lungs, and brain by plaque assay as previously described^39,40^. Total leukocytes in the BALF were firstly enumerated with a total cell count using a hemocytometer, after which cells were deposited onto glass slides using a cytospin. Slides were then stained using a Quick Dip Stain Kit (Modified Giemsa Stain) protocol as per the manufacturer’s instructions (POCD Scientific, Australia) and differential cell counts obtained.

### Statistical analysis

Statistical analysis was performed in GraphPad Prism 9 software (GraphPad, California, USA). Prior to analysis data the assumption of normal distribution was assessed using the F-test. One- or Two-way ANOVA was performed where appropriate, with post-hoc Tukey, Holm-Sidak’s, or Sidak’s multiple comparisons test *p* < 0.05 (*), *p* < 0.005 (**), *p* < 0.0005 (***). Where otherwise appropriate, an unpaired two-tailed T-test was performed, *p* < 0.05 (*), *p* < 0.005 (**), *p* < 0.0005 (***). The datasets generated during and/or analyzed during the current study are available from the corresponding author on reasonable request.

## Supplementary information

Author checklist

Supplementary information
