# Supplementary material for: Mucosal immunization with a delta-inulin adjuvanted recombinant spike vaccine elicits lung-resident immune memory and protects mice against SARS-CoV-2
Source: Mucosal Immunol. 2022 Nov 21;15(6):1405–15. doi: 10.1038/s41385-022-00578-9 (PMC9676795; doi:10.1038/s41385-022-00578-9)
Supplement: Supplementary file 2 — Supplementary information [file 41385_2022_578_MOESM2_ESM.pdf]

Mucosal immunization with a delta-inulin adjuvanted recombinant spike vaccine elicits lung-resident immune memory and protects mice against SARS-CoV-2

SUPPLEMENTARY INFORMATION

Erica L. Stewart[1], Claudio Counoupas[1,2,5], Matt D. Johansen[3], Duc H. Nguyen[3], Stefan Miemczyk[3], Nicole G. Hansbro[3], Kia Ferrell[1], Anneliese Ashhurst[1,2,5], Sibel Alca[1], Megan Steain[1,5], Caroline Ashley[1], Warwick J. Britton[2,4], Philip M. Hansbro[3], Nikolai Petrovsky[6], James A. Triccas[1,5]

1. School of Medical Sciences, Faculty of Medicine and Health, Charles Perkins Centre, The University of Sydney, NSW, Australia
2. Tuberculosis Research Program at the Centenary Institute, The University of Sydney, Sydney, NSW, Australia
3. Centre for Inflammation, Centenary Institute and University of Technology Sydney, Faculty of Science, School of Life Sciences, Sydney, NSW, Australia
4. Department of Clinical Immunology, Royal Prince Alfred Hospital, Sydney, NSW, Australia
5. Sydney Institute for Infectious Diseases and the Charles Perkins Centre, The University of Sydney, Camperdown, NSW, Australia
6. Vaxine Pty Ltd., Bedford Park, Adelaide 5042, SA, Australia

Corresponding author: James A Triccas

Email: jamie.triccas@sydney.edu.au

Supplementary Table 1: Fluorescent-conjugated monoclonal antibodies used for flow cytometric analysis

**Antibody**	**Clone**	**Manufacturer**
Anti-mouse B220 BUV395	RA3-6B2	BD Horizon
Anti-mouse CD103 BV786	M290	BD Horizon
Anti-mouse CD11c PE/Cy7	HL3	BD Pharmingen
Anti-mouse CD16/CD32 purified	2.4G2	BD Pharmingen
Anti-mouse CD19 BV786	1D3	BD Horizon
Anti-mouse CD4 AF700	RM4-5	BD Pharmingen
Anti-mouse CD4 APC	GK1.5	eBioscience
Anti-mouse CD44 BV605	IM7	BD Horizon
Anti-mouse CD44 FITC	IM7	BD Pharmingen
Anti-mouse CD62L APC/Cy7	MEL-14	BioLegend
Anti-mouse CD69 FITC	H1.2F3	BD Pharmingen
Anti-mouse CD8a Pacific Blue	53-6.7	BD Pharmingen
Anti-mouse GATA3 BUV395	L50-823	BD Biosciences
Anti-mouse RorγT PECF594	Q31-378	BD Horizon
Anti-mouse Siglec-F BV711	E50-2440	BD Biosciences
Anti-mouse T-bet PerCpCy5.5	4B10	BioLegend
Anti-mouse IL-5 APC	TRFK5	BD Pharmingen
Anti-mouse IL-17A BV421	TC11-18H10	BD Biosciences
Anti-mouse IL-10 BV605	JES5-16E3	BD Horizon
Anti-mouse TNF PerCPCy5.5	MP6-XT22	BD Pharmingen
Anti-mouse IL-2 PE	JES6-5H4	BD Pharmingen
Anti-mouse IFN-γ PE-Cy7	XMG1.2	BD Pharmingen

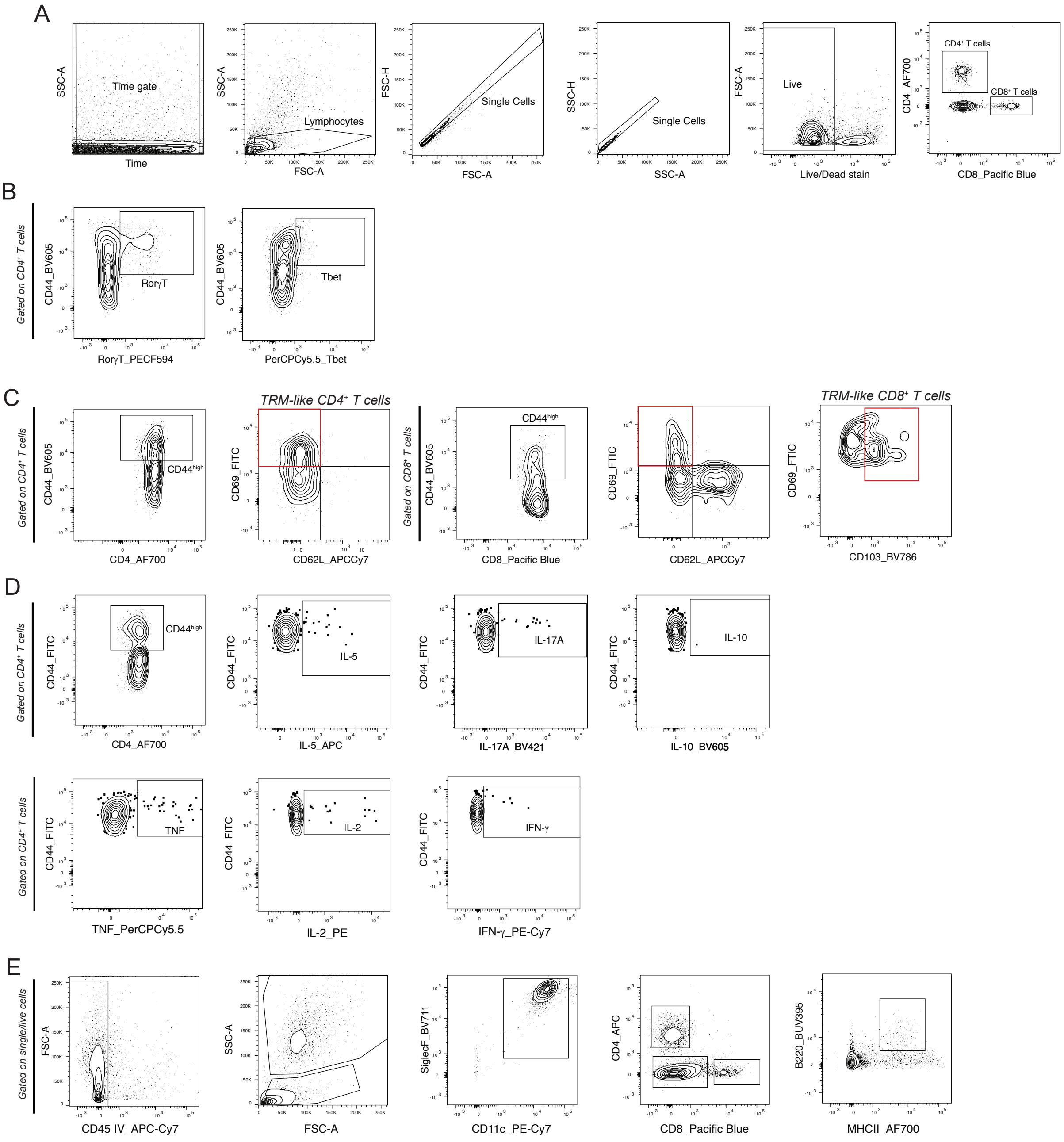

Supplementary Figure 1: $CD4^+$ and $CD8^+$ T cells were gated in the lungs and lymph nodes as per (A), first on a time gate, then FSC-A and SSC-A singlets to exclude debris, live cells and then gated on CD4 and CD8 expression. $CD4^+$ T cell transcription factor expression was gated as per (B). (C) $CD4^+$ TRM-like cells were defined as $CD44^+CD69^+$ and $CD62L^-$; $CD8^+$ TRM-like cells were defined as $CD44^+CD69^+$CD103 and $CD62L^-$. $CD4^+$ T cell cytokine expression after restimulation was defined as in the gating in (D) - cells were gated on $CD44^{high}$ then on their respective cytokines. (E) BALF cells were gated as live, single cells, then as intravascular negative. Alveolar macrophages were gated by size then expression of CD11c and SiglecF. Lymphocytes were gated by size followed by CD4 and CD8 expression; B cells were gated as $CD4^-CD8^-B220^+MHCII^+$.

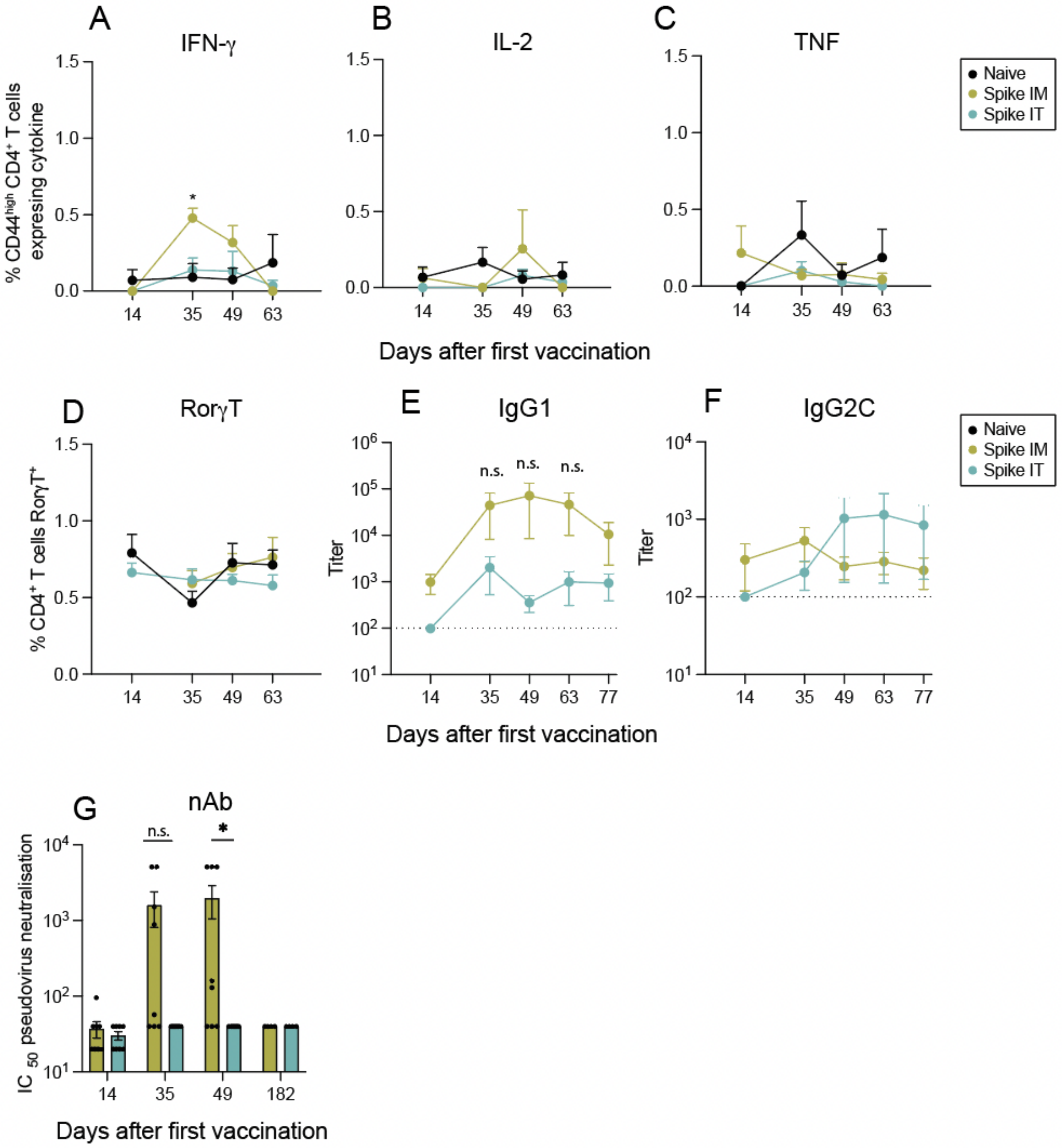

Supplementary Figure 2: C57BL/6 mice were vaccinated as in Figure 1, shown here are only the spike alone controls. At time points post prime and boost, mice were bled and PBMCs were restimulated with peptides, then stained intracellularly for cytokine production (IFN-γ, IL-2, IL-17A and TNF) measured by flow cytometry (A-C). Unstimulated PBMCs were also stained intracellular for transcription factor expression (RorγT) (D), measured by flow cytometry. Antibody titers of plasma were determined by ELISA (E-F, H-I), and neutralising antibody titers were determined in plasma using a pseudovirus assay (G). IgA titers were measured for plasma (diluted 1:20) two weeks post second immunization (H) and in BALF (diluted 1:5) eight weeks post second immunization (I). A-G depict pooled or representative data from more than two experiments, H and I depicts data from a single experiment, with four or five mice per group. For A-G, the significance between groups was determined via a two-way ANOVA with post-hoc Sidak's multiple comparison's test, $p<0.05$ (*), $p<0.005$ (**), $p<0.0005$. (***).

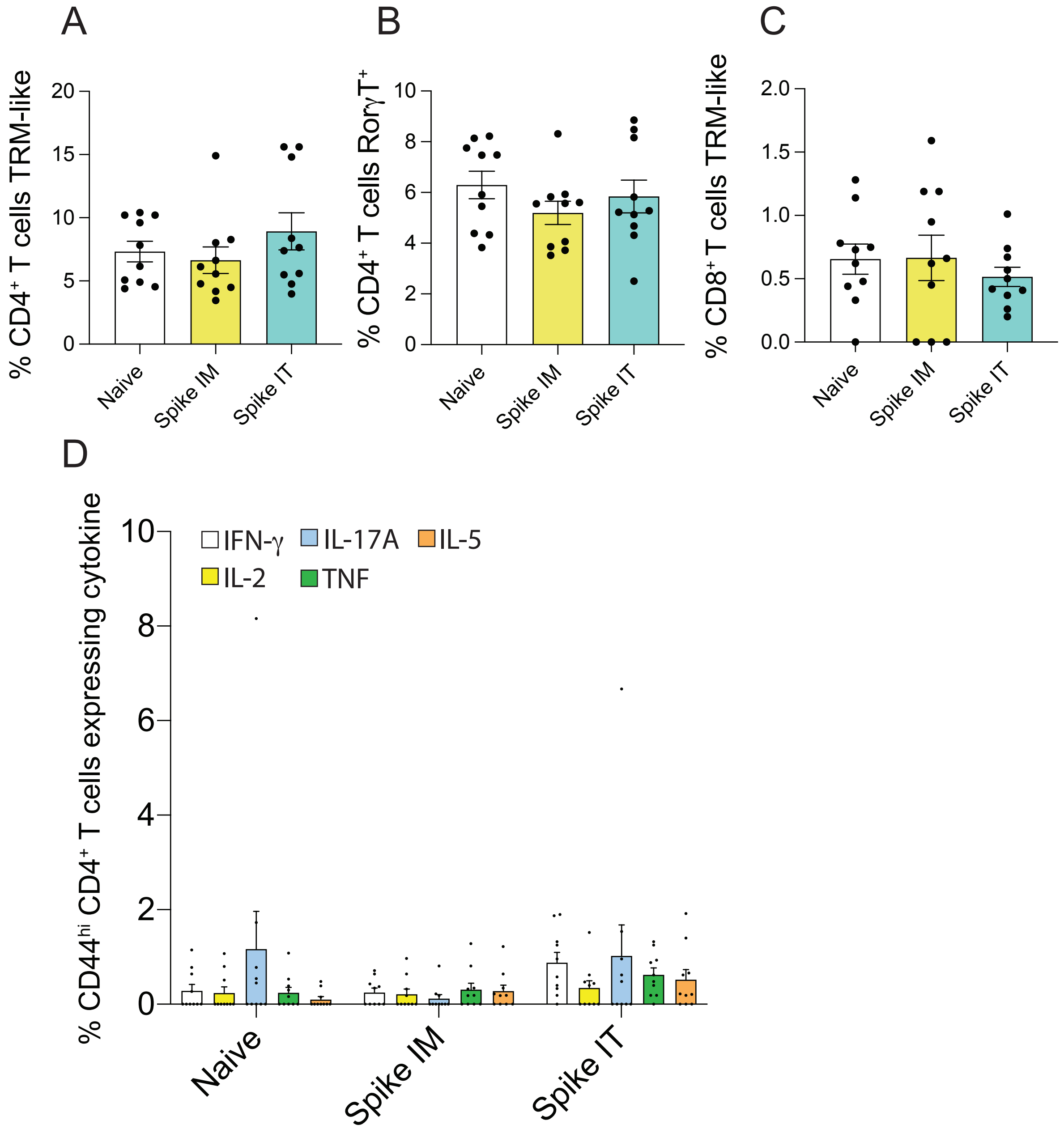

Supplementary Figure 3: C57BL/6 mice were vaccinated as in Figure 1, shown here are only the spike alone controls. Lungs and lymph nodes were collected eight weeks after the final vaccination for flow cytometric analysis. Shown here are spike alone controls compared with naïve. $CD4^+$ (A) and $CD8^+$ (C) T cells in the lungs were characterised for T resident memory (TRM-like) marker expression ($CD44^+CD69^+$, $CD62L^-$, or $CD44^+CD103^+CD69^+CD62L^-$ respectively) by flow cytometry. B) $CD4^+$ T cells were measured for RorγT expression. D) Lung cells were also restimulated with spike peptides and stained intracellularly for cytokines (IFN-g, IL-2, IL-17A, TNF and IL-5) (E). Graphs depict pooled data from more than two experiments, with four or five mice per group. The significance between groups was determined via one or two-way ANOVA with post-hoc Tukey test respectively, $p<0.05$ (*), $p<0.005$ (**), $p<0.0005$. (***).

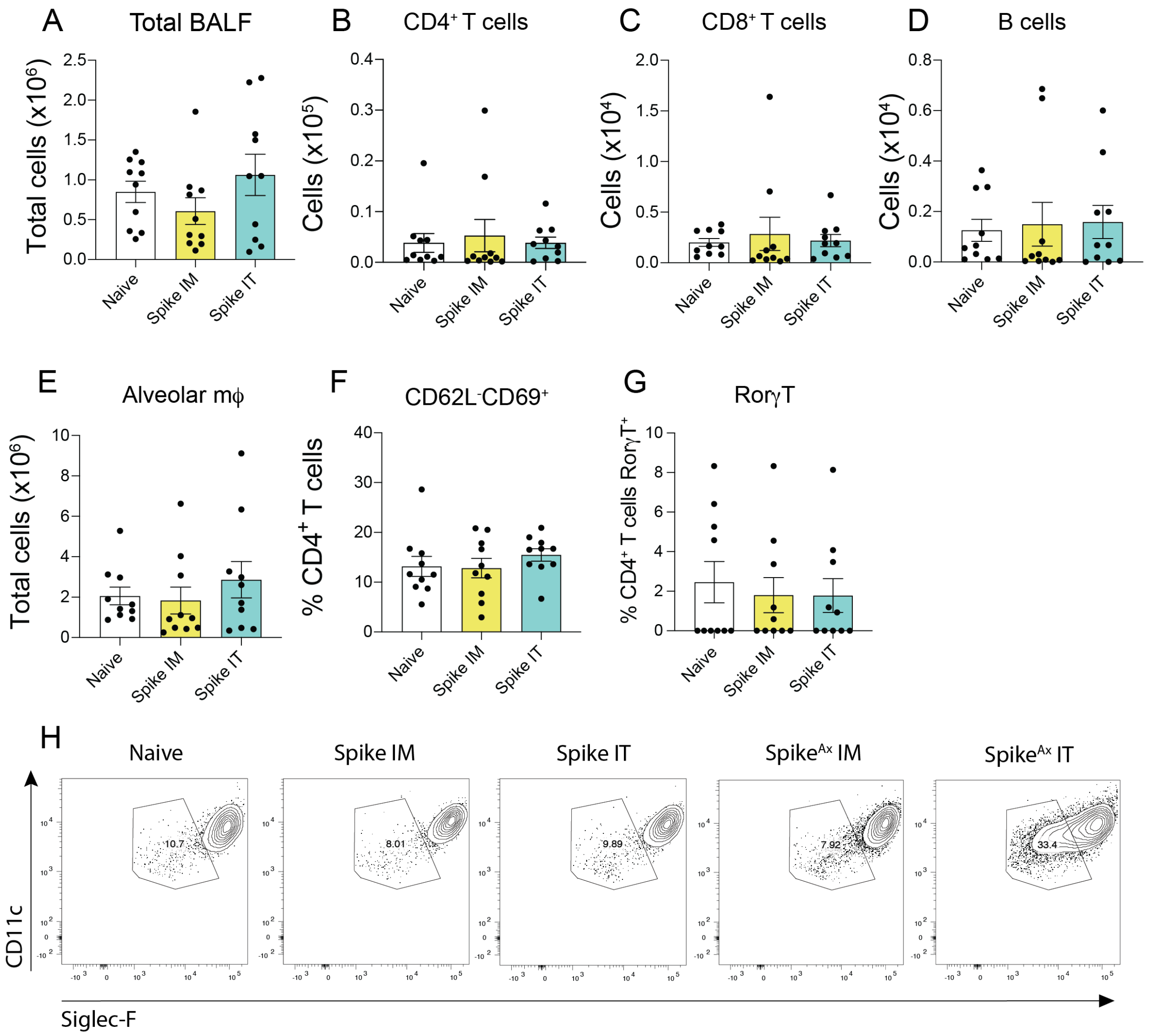

Supplementary Figure 4: C57BL/6 mice were vaccinated as in Figure 1, shown here are only the spike alone controls. Bronchoalveolar lavage fluid (BALF) was collected eight weeks after the final vaccination for flow cytometric analysis. A) Total BALF cell number. Total number of $CD4^+$, $CD8^+$ T cells and B cells (B-D), and alveolar macrophages (E) in the BALF. F) Phenotype of $CD4^+$ T cells in the BALF G) RorγT expression on $CD4^+$ T cells in the BALF. H) SiglecF expression on alveolar macrophages in the airways. Graphs depict pooled data from two experiments, with four or five mice per group. FACS plots are representative of more than two experiments. The significance between groups was determined via a one-way ANOVA with post-hoc Tukey test $p<0.05$ (*), $p<0.005$ (**), $p<0.0005$. (***).

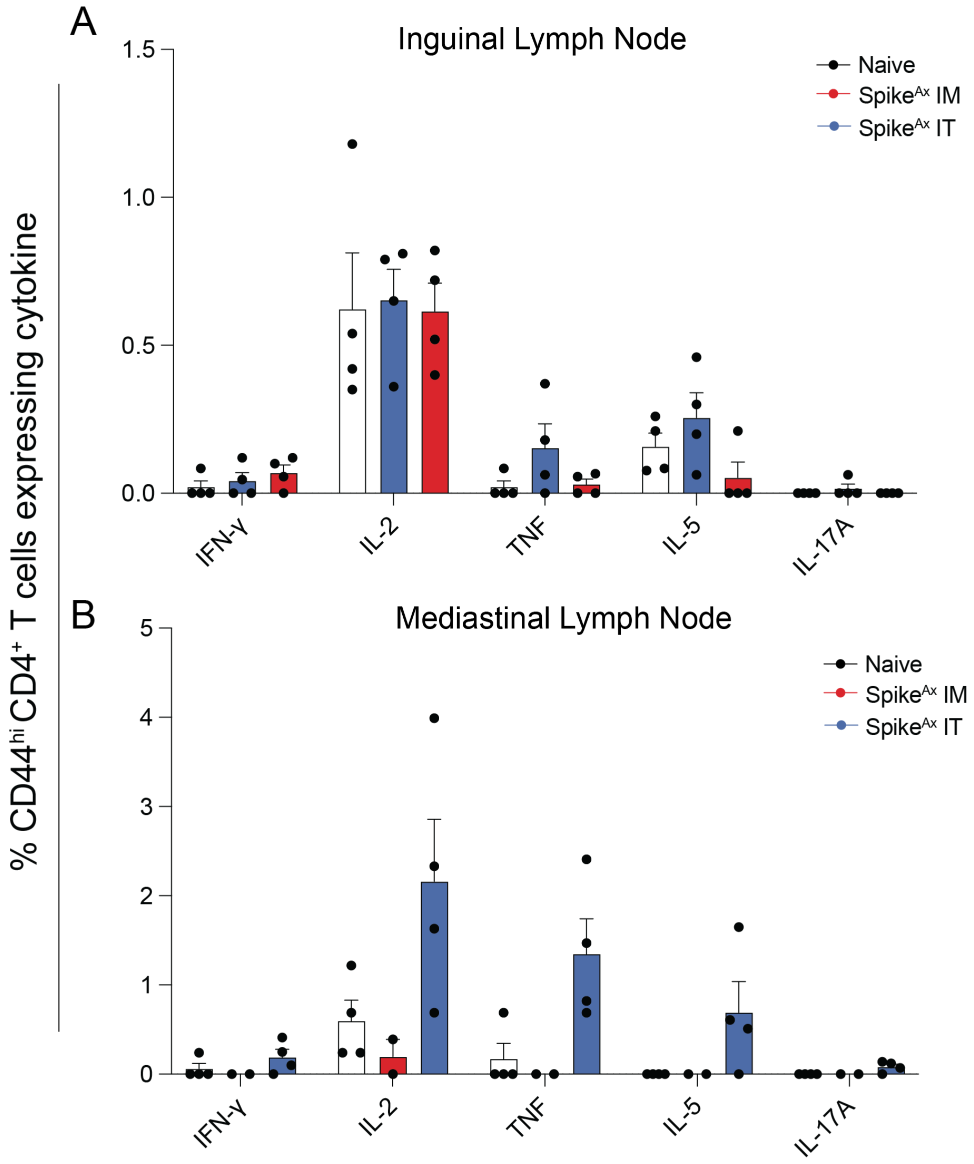

Supplementary Figure 5: C57BL/6 mice were vaccinated as in Figure 1, shown here are only the spike alone controls. Lymph nodes were collected from $Spike^{Ax}$ animals eight weeks after the final vaccination and restimulated with spike peptides. After restimulation, IFN-γ, IL-2, IL-17A, TNF and IL-5 were measured using flow cytometry. Cells from the inguinal lymph node (A) and mediastinal lymph node (B) were gated on $CD4^{+}$ $CD44^{hi}$ then cytokine positive. Graphs are representative of more than two experiments with four or five mice per group. The significance between groups was determined via a two-way ANOVA with post-hoc Tukey test $p<0.05$ (*), $p<0.005$ (**), $p<0.0005$. (***).

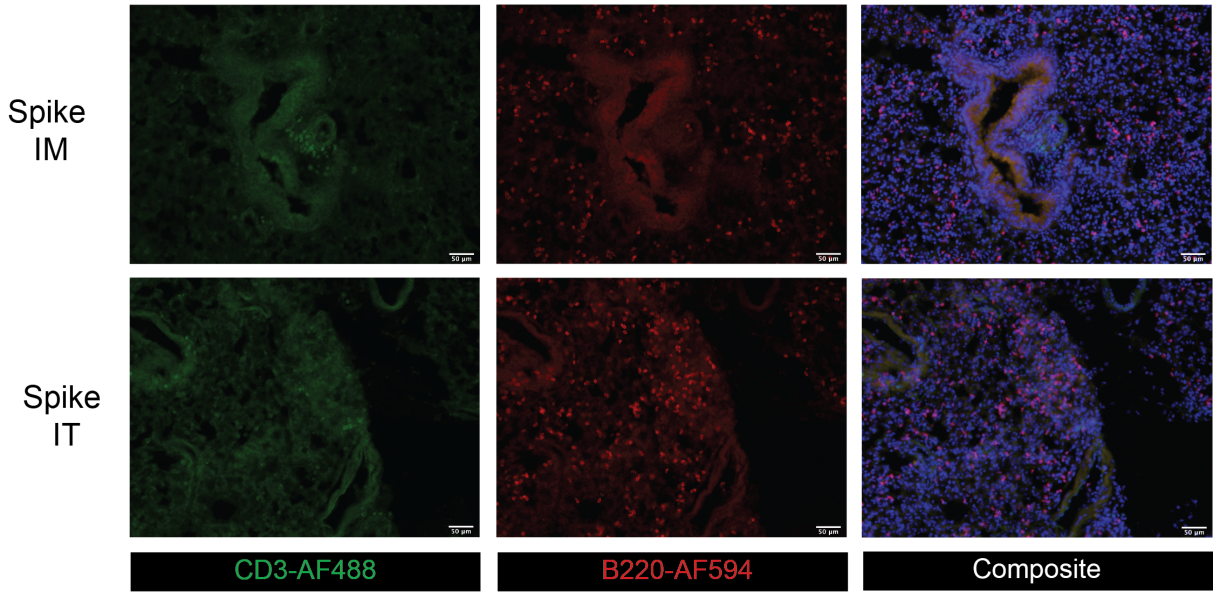

Supplementary Figure 6: C57BL/6 mice were vaccinated as in Figure 1, shown here are only the spike alone controls. Eight weeks after booster vaccination lung lobes were collected for imaging. Shown here are spike alone controls. Lobes were stained with anti CD3-AF488 (green), anti B220-AF594 (red) and DAPI (blue). Images are representative of four mice from each group, from one experiment.

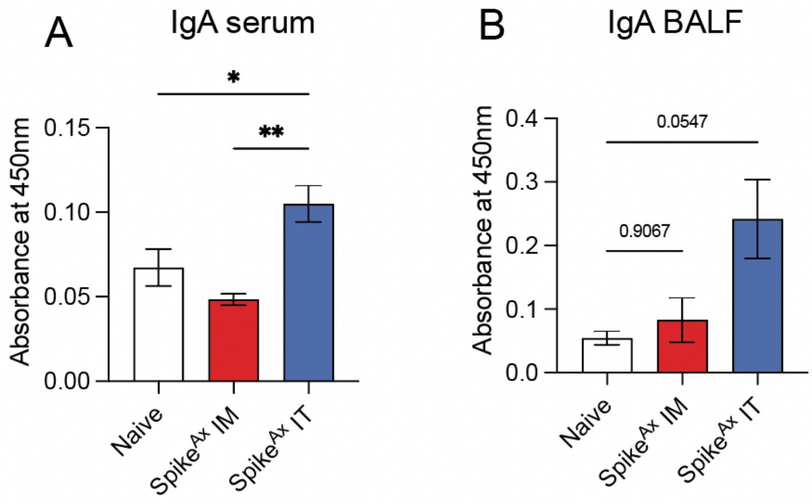

Supplementary Figure 7: C57BL/6 mice were vaccinated as in Figure 1. At time points post prime and boost, mice were bled and antibody titers of plasma were determined by ELISA. IgA titers were measured for plasma (diluted 1:20) two weeks post second immunization (A) and in BALF (diluted 1:5) eight weeks post second immunization (B). A and B depicts data from a single experiment, with four or five mice per group. The significance between groups was determined via a one-way ANOVA with post-hoc Tukey's multiple comparison's test, p<0.05 (*), p<0.005 (**), p<0.0005. (***).
